# SLPI suppresses hepatocellular carcinoma progression via endoplasmic reticulum stress induced apoptosis

**DOI:** 10.7150/ijbs.65676

**Published:** 2022-01-01

**Authors:** Jie Sun, Jinfan Li, Zhen Wu, Yuwan Liang, Rong Duan, Mengsha Zheng, Jing Wang, Derun Kong

**Affiliations:** 1 Department of Pathology, Second Affiliated Hospital of Zhejiang University School of medicine, Jiefang Road 88, Hangzhou 310009, Zhejiang Province, China.; 2 Department of Gastroenterology, The First Affiliated Hospital of Anhui Medical University, Jixi Road 218, Hefei, 230032, China.; 3 Department of Gastroenterology, Fuyang Hospital of Anhui Medical University, Huangshan Road 99, Fuyang, 236000, China.; 4 Department of Ultrasound in Medicine, Second Affiliated Hospital of Zhejiang University School of medicine, Jiefang Road 88, Hangzhou 310009, Zhejiang Province, China.; 5 Department of Hygiene Inspection and Quarantine, School of Public Health, Anhui Medical University, Meishan Road 81, Hefei 230022, Anhui Province, China.

**Keywords:** SLPI, Secretory leukocyte protease inhibitor, Hepatocellular carcinoma, Endoplasmic reticulum stress, Apoptosis, MAPK.

## Abstract

Hepatocellular carcinoma (HCC) is one of the most common malignancies worldwide. Secretory leukocyte protease inhibitor (SLPI) has been reported to function as a regulatory factor in several cancers. However, its biological functions and underlying mechanisms in HCC remain to be uncovered. Here, we aimed to explore the effect of SLPI in HCC. In our study, we found that the mRNA and protein expression levels of SLPI were significantly down-regulated in HCC tissues and hepatoma cell lines and low level of SLPI predicted worse survival in our HCC cohorts. In term of function, silencing of SLPI markedly promoted whereas overexpression SLPI suppressed proliferation, migration and invasion capabilities of HCC cells *in vitro*, and ectopic expression of SLPI inhibited the tumorigenicity of HCC cells *in vivo*. Mechanistic studies demonstrated that SLPI played a protective role in HCC progression via activating endoplasmic reticulum stress (ER stress)-mediated apoptosis of hepatoma cells, which could be regulated by MAPK signaling pathways. In summary, our findings highlight that SLPI could serve as a potential prognostic biomarker and putative tumor suppressor by enhancing ER stress-induced apoptosis in HCC cells mediated by MAPK signaling pathways, which provides new insights into promising therapeutic targets for HCC treatment.

## Introduction

Hepatocellular carcinoma (HCC) is one of the most common cancers worldwide 1. In developed countries, deaths caused by HCC occupy the sixth place among male cancer-related deaths. However, in developing countries including China, deaths caused by HCC rank second. There are about one million new cases of HCC in the world annually, and more than three-quarters of patients will be dead, especially more than half of the incidence and mortality of HCC come from China 2. Although the latest statistics in 2020 show that the incidence of HCC has decreased, the growth rate of HCC has been the first rate, about 2%-3% from 2007 to 2016 3. Although many clinical interventions have been improved slightly, the survival status of HCC patients is still not ameliorated significantly. It is imperative to elucidate more novel molecular changes that are beneficial to the treatment and diagnosis of HCC.

Most HCC cases develop with a significant feature which were usually based on hepatic cirrhosis and often associated with chronic hepatitis B or C virus infection 4. In fact, only 50% of HCC-related deaths are virus-dependent in developed countries. Among the other half, most of the patients are non-alcoholic steatohepatitis, which is considered to be a major risk factor for HCC 5. And recent evidence suggests that ER stress plays a pivotal role in the progression of non-alcoholic steatohepatitis induced HCC 6-8. Therefore, it will be of paramount significance to enhance ER stress-induced apoptosis of HCC cells in order to make an important contribution to the therapeutic strategies for HCC.

Secretory secretory leukocyte protease inhibitor (SLPI) is mainly expressed in epithelial cells 9. SLPI, a member of Whey acidic protein family, could mediate a variety of biological processes including cell proliferation and apoptosis, repairing reaction and immune response, with crucial roles in anti-protease, anti-inflammatory, anti-bacterial and anti-virus 10, 11. The existing researches have shown SLPI could serve as a tumor regulator to mediate the progression and prognosis of a wide range of cancers. SLPI has been demonstrated to be down-regulated in nasopharyngeal carcinoma, bladder cancer, prostate cancer and breast cancer 12-14. Moreover, the expression levels of SLPI were correlated with tumor growth and invasion capacities 15. Further researches has found that SLPI is involved in the process of TNFα-induced monocyte apoptosis via downregulating the enzymatic activity of caspase-3/7 16.The studies above indicated that SLPI could take part in regulation of cell apoptosis which serves as a key regulator in the progression of various tumors. However, whether SLPI was involved in the progression of HCC remained a mystery. With the consideration of these, we here hypothesized that SLPI may affect the progression of HCC via regulating apoptosis of hepatoma cells.

In the present study, we detected that SLPI was expressed at a lower level in tumor liver tissues compared with non-tumor liver tissues which was closely correlated with poor clinical outcome of HCC patients. By manipulating the expression of SLPI in cultured hepatoma cells and xenografted tumor tissue, we observed that SLPI could inhibit proliferation, migration and invasion capacities of HCC cells. Further experiments confirmed the pivotal role of the MAPK signaling pathways in mediating effect of ER stress-induced apoptosis on hepatocellular carcinoma cell proliferation, migration, and invasion. Based on our findings, SLPI may serve as a novel therapeutic target for HCC.

## Results

### SLPI was down-regulated in HCC tissues and cell lines

**Figure 1:**
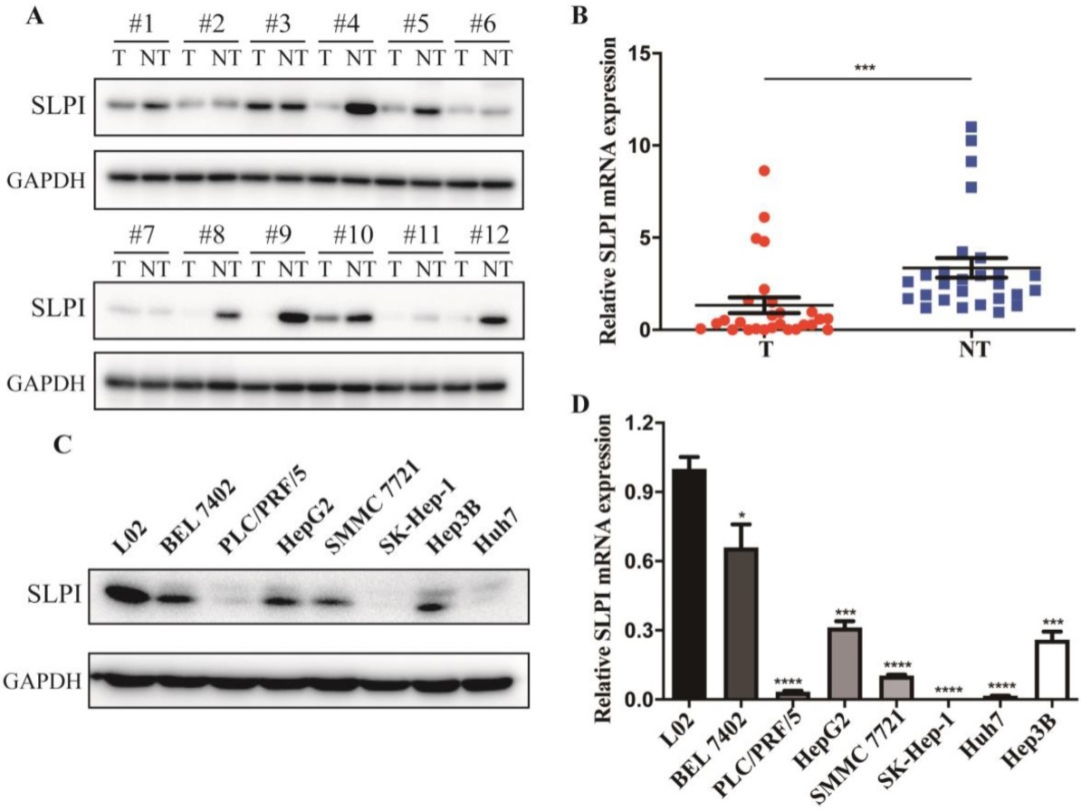
** SLPI expression is decreased in HCC. A.** Western Blotting (WB) analysis of SLPI in HCC tissues (T) and matched adjacent noncancerous tissues (NT). Levels of SLPI quantified by optical density and normalized to GAPDH levels in the same samples are shown below the WB results relative to levels in NT.** B.** RT-PCR analysis of SLPI in 27 paired T and their ANT. The average SLPI mRNA expression was normalized to the expression of GAPDH. **C.** SLPI protein expression was detected in L02 and HCC cell lines by WB analysis. Levels of SLPI quantified on the basis of optical density and normalized to GAPDH levels in the same samples are shown below the WB results relative to levels in L02 cells. **D.**SLPI mRNA expression was detected in L02 and HCC cell lines by using RT-PCR analysis. Three independent experiments were conducted in each assay. *p<0.05, **p<0.01, ***p<0.001, ****p<0.0001.

The first set of experiments aimed to investigate the expression of SLPI in human HCC tissues and cell lines. We detected the protein and mRNA expression of SLPI in 12 tested liver tumor tissues (T) and the matched adjacent noncancerous tissues (ANT). The WB results revealed that SLPI protein expression level was markedly down-regulated in T compared with the matched ANT (Figure 1A). Decreased mRNA expression level of SLPI in T was further confirmed by using RT-PCR analysis (Figure 1B). Furthermore, we performed WB and RT-PCR to investigate the expression of SLPI in seven HCC cell lines and normal liver cells. We found that the SLPI expression levels were reduced in seven HCC cell lines compared with L02 cells (Figure 1C and 1D). These results were strongly indicative of down-regulation of SLPI in HCC.

### Low SLPI expression in HCC tissues correlated with poor outcomes of patients

**Figure 2:**
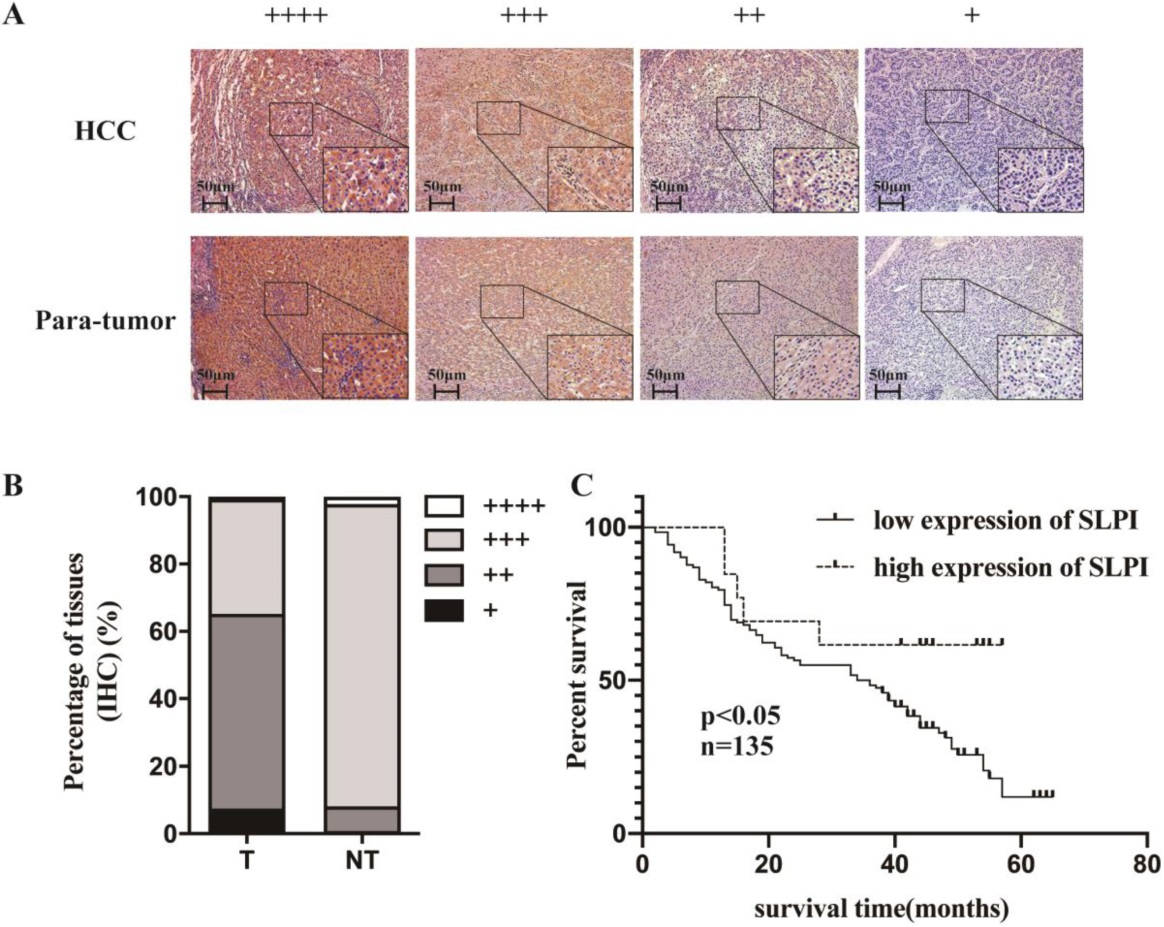
**Low SLPI expression in HCC tissues is correlates with poor patient survival. A. B.** Expression levels of SLPI in HCC tissues compared with their paired para-tumor tissues as determined through IHC staining. **C.** Overall survival curves for all 135 patients with HCC stratified by high and low expression of SLPI.

IHC was applied to explore the frequency of SLPI down-regulation in 135 paraffin-embedded, archived HCC tissues (Table 1). Table 1 shows that SLPI expression was at low levels in 83 samples (83/135, 61.5%). These results showed that the degree of SLPI protein expression varies in the different samples and were generally down-regulated in HCC tissues compared with para-tumor tissues (Figure 2A, 2B). The results of clinicopathological analysis were summarized in Table 1 and table 2. Kaplan-Meier survival analysis revealed that the overall survival rate was lower in the low SLPI group (Figure 2C, p < 0.05). Univariate Cox regression analysis presents that low expression of SLPI was associated with a higher risk of death in HCC patients. Multivariate Cox regression analysis provides that SLPI expression level was a predictive indicator for overall survival (P =0.042) for HCC patients (Table 3). Collectively, the above findings indicated that down-regulation of SLPI was closely correlated with poor survival in patients with HCC.

### SLPI inhibited HCC cell proliferation, migration and invasion *in vitro*

**Figure 3:**
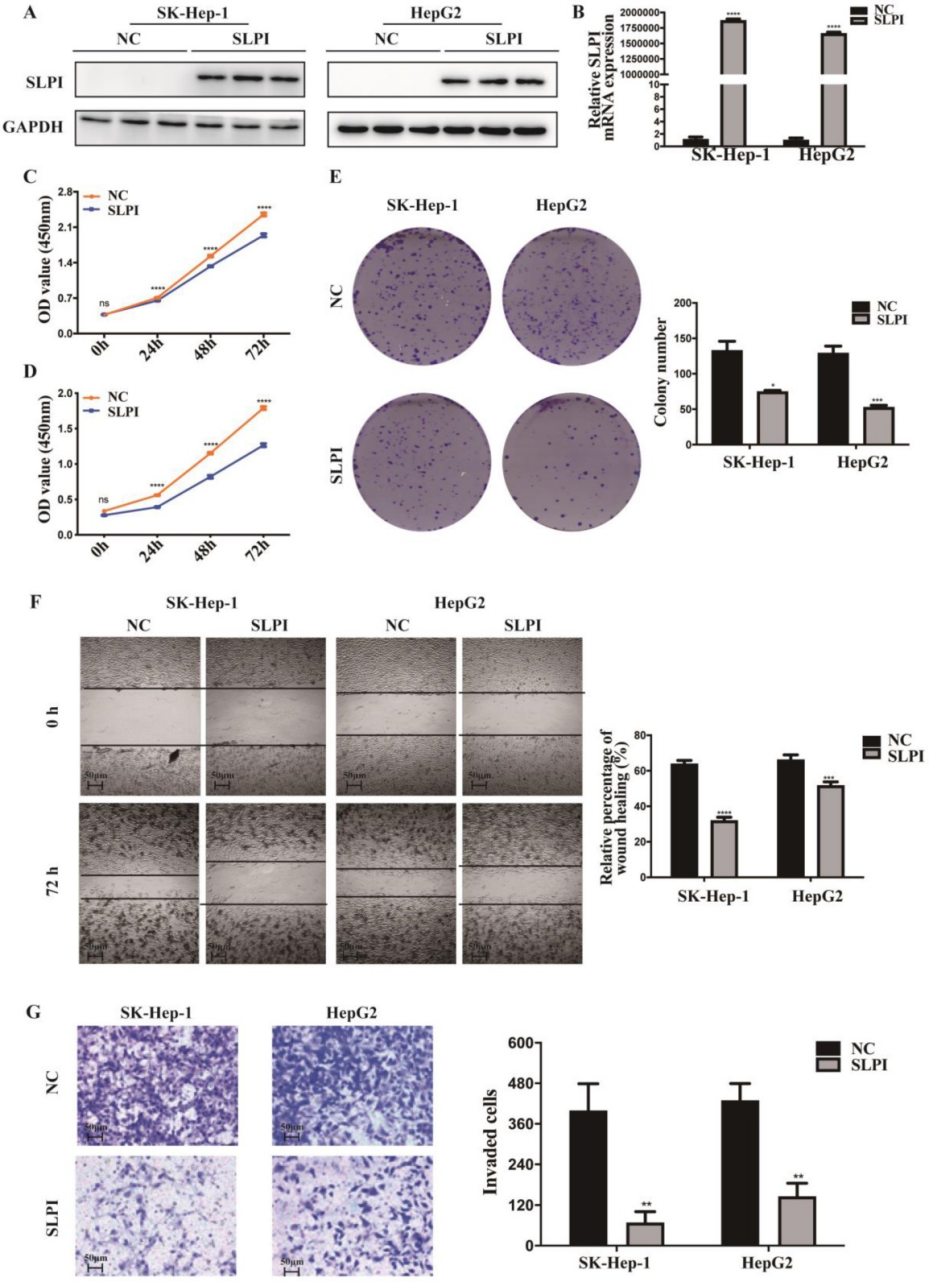
** SLPI overexpression inhibited HCC cell proliferation, migration and invasion *in vitro*. A. B.**WB and RT-PCR analysis of SK-Hep-1 and HepG2 cells infected with SLPI and negative control (NC) lentivirus. Levels of SLPI quantified on the basis of optical density and normalized to GAPDH levels in the same samples are shown below the WB results relative to levels in NC cells.** C. D.** CCK8 assays were performed to investigate the proliferation of SK-Hep-1 and HepG2 cells infected with SLPI lentivirus. **E.** colony formation assays were performed to determine the colonies of cells with SLPI overexpression.** E.** Wound healing assays were performed to examine the cell migration of SLPI overexpression. **F.** Transwell assays were completed to explore the effects of SLPI overexpression on cell invasion. *p<0.05, **p<0.01, ***p<0.001, ****p<0.0001.

**Figure 4:**
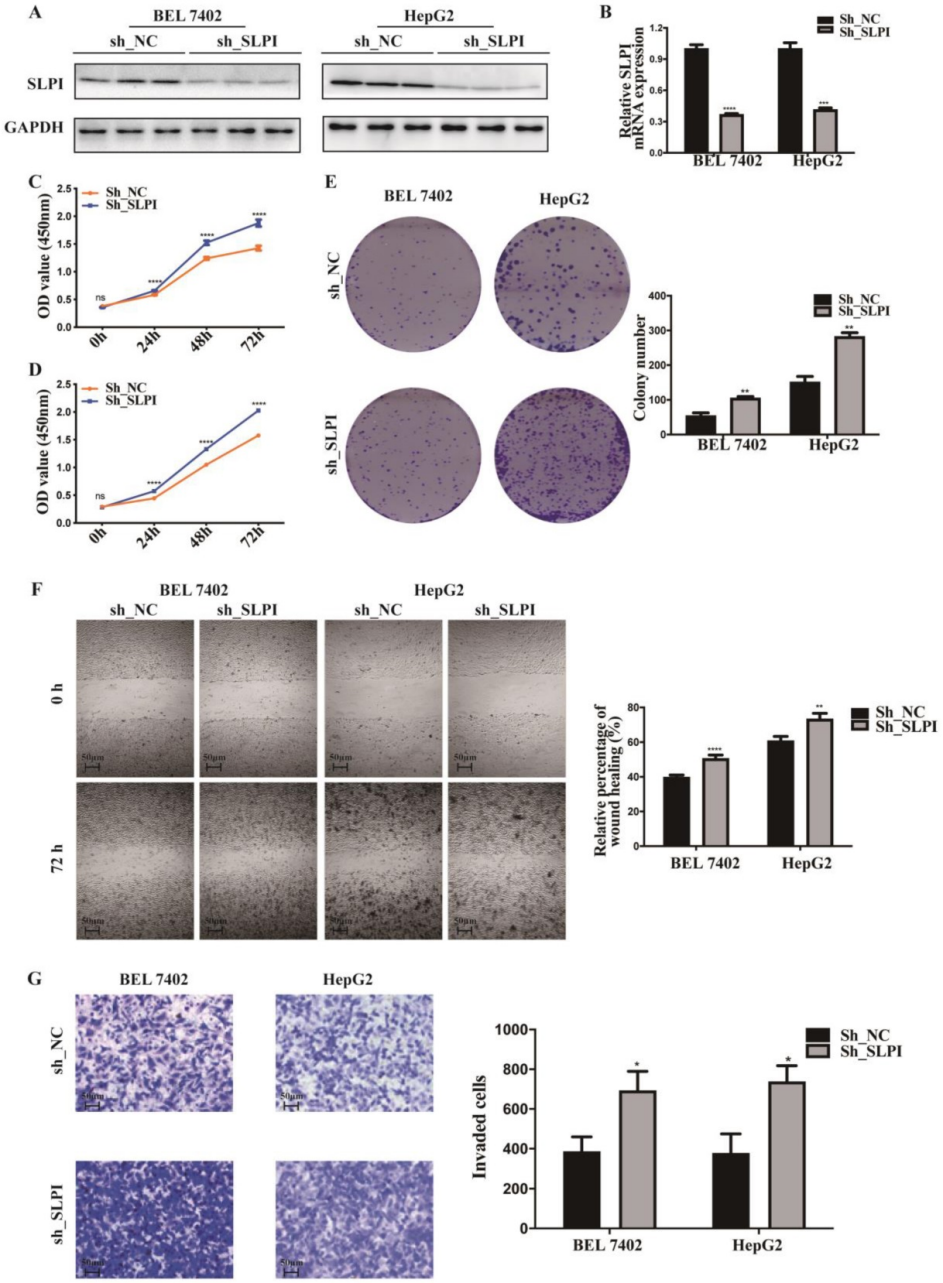
** SLPI knock-down promoted HCC cell proliferation, migration and invasion *in vitro*. A. B.**WB and RT-PCR analysis of BEL 7402 and HepG2 cells infected with SLPI shRNA and negative control (NC) lentivirus. Levels of SLPI quantified on the basis of optical density and normalized to GAPDH levels in the same samples are shown below the WB results relative to levels in NC cells.** C. D.** CCK8 assays were performed to investigate the proliferation of BEL 7402 and HepG2 cells infected with SLPI shRNA lentivirus. **E.** Colony formation assays were performed to determine the colonies of cells with SLPI knockdown.** E.** Wound healing assays were performed to examine the cell migration of SLPI down-regulation. **F.** Transwell assays were completed to explore the effects of SLPI knockdown on cell invasion. *p<0.05, **p<0.01, ***p<0.001, ****p<0.0001.

In figure 1, we acquired the protein and mRNA expression levels of SLPI in seven HCC cell lines and L02 cells. According to these results, moderate SLPI-expressing HepG2 and low SLPI-expressing SK-Hep-1 were chosen. Then we stably overexpressed SLPI in the two cell lines by lentivirus (Figure 3A, 3B). Similarly, moderate SLPI-expressing HepG2 and high SLPI-expressing BEL 7402 were seleced to knockdown the endogenous expression of SLPI (Figure 4A, 4B).

To explore the function role of SLPI in HCC, we performed cell proliferation, migration and invasion assays. CCK-8 assay showed that cell proliferation ability was inhibited in both SK-Hep-1 and HepG2 cells by up-regulation of SLPI (Figure 3C, 3D). The results of colony formation assay confirmed that SLPI overexpression impaired proliferating function of SK-Hep-1 and HepG2 cells (Figure 3E). Wound healing assay indicated that SLPI-overexpressing SK-Hep-1 and HepG2 cells delayed wound healing (Figure 3F). In transwell assay, overexpression of SLPI suppressed the invasion of SK-Hep-1 and HepG2 cells (Figure 3F). Moreover, the effects of SLPI knockdown were further confirmed. In CCK-8 and colony formation assays, down-regulation of SLPI promoted proliferation ability of BEL 7402 and HepG2 cells (Figure 4C, 4D, 4E). Results from wound healing assay presented that down-regulation of SLPI enhanced migration ability of BEL 7402 and HepG2 cells (Figure 4F). As shown in Figure 4G, SLPI deficiency increased invaded BEL 7402 and HepG2 cells (Figure 4G). These results suggested that SLPI could be a pivotal player in mediating malignant biological behaviors of HCC.

### SLPI facilitated apoptosis of HCC cells

**Figure 5:**
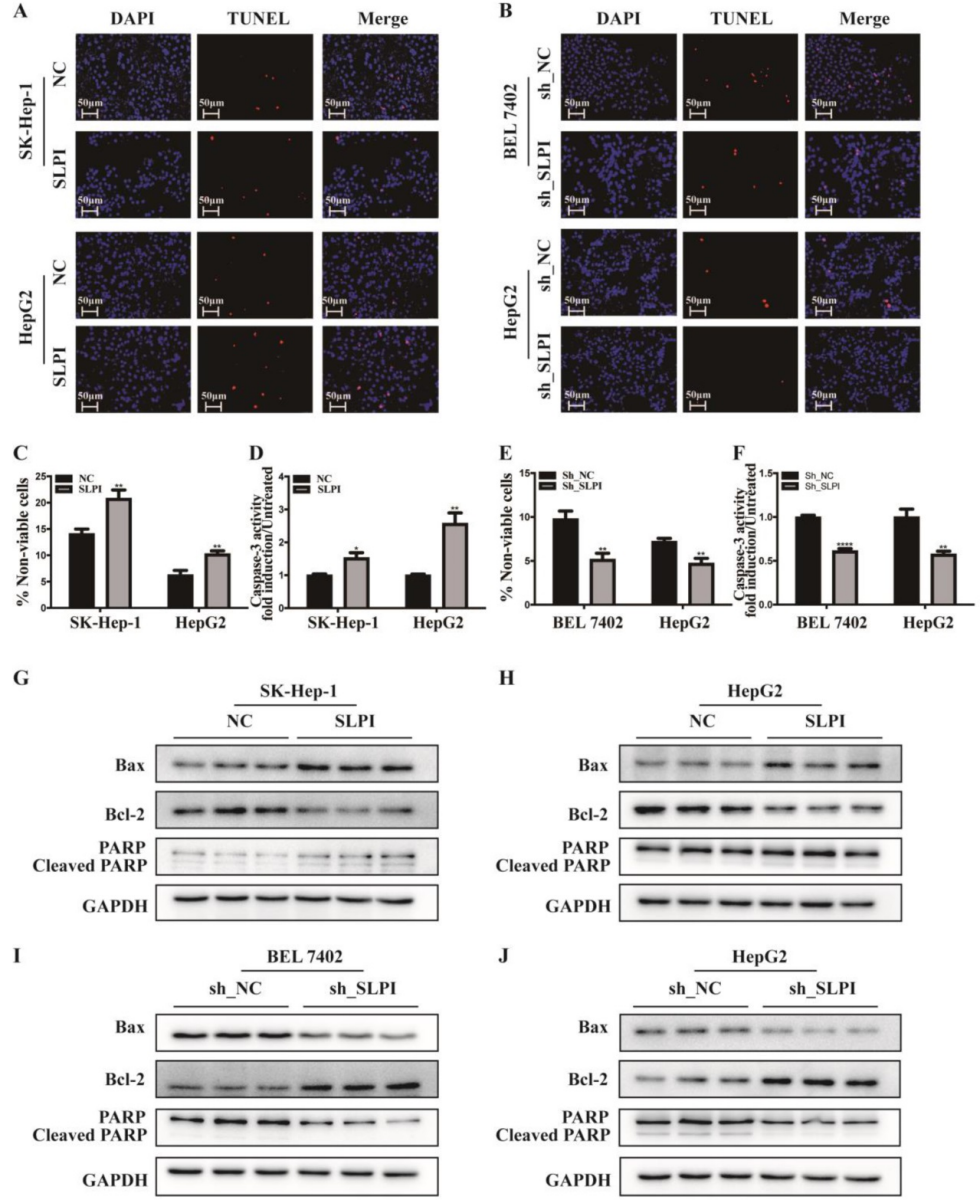
** SLPI regulated HCC apoptosis. A.** TUNEL assays were performed to determine the apoptosis of SK-Hep-1 and HepG2 cells infected with SLPI and negative control (NC) lentivirus. **B.** TUNEL assays were performed to examine the apoptosis of BEL 7402 and HepG2 cells with SLPI knockdown. **C.** Trypan blue staining analysis were performed to investigate the apoptosis of SK-Hep-1 and HepG2 cells with SLPI overexpression. **D.** Caspase-3 activity analysis were performed to determine the apoptosis of SK-Hep-1 and HepG2 cells with SLPI up-regulation. **E.** Trypan blue staining analysis were performed to examine the apoptosis of BEL 7402 and HepG2 cells with SLPI knockdown. **F.** Caspase-3 activity analysis were performed to determine the apoptosis of BEL 7402 and HepG2 cells with SLPI down-regulation. **G. H.** WB analysis of Bax, Bcl2, cleaved PARP and PARP in SK-Hep-1 and HepG2 cells with SLPI overexpression. **I. J.** WB analysis of Bax, Bcl2, cleaved PARP and PARP in BEL 7402 and HepG2 cells with SLPI knockdown.

TUNEL assay, trypan blue staining and caspase-3 activity determination were performed to validate the biological function of SLPI in apoptosis of hepatoma cells. The results of TUNEL assay presented that SLPI-overexpressing SK-Hep-1 and HepG2 cells appeared a higher rate of apoptosis (Figure 5A). However, the trends in SLPI-underexpressing BEL 7402 and HepG2 cells were going in the other direction (Figure 5B). Trypan blue staining demonstrated that apoptosis in HCC cells was induced by up-regulation of SLPI and inbihited by down-regulation of SLPI (Figure 5C, 5E). And the results of caspase-3 activity determination confirmed that upregulation of SLPI promoted apoptotic response in SK-Hep-1 and HepG2 cells (Figure 5D), whereas knockdown of SLPI impaired apoptosis in BEL 7402 and HepG2 cells (Figure 5F).

Furtherly we investigated the effect of SLPI on apoptosis associated proteins like Bax, Bcl2 and PARP. The results of WB revealed that the expression levels of Bax and PARP, cleaved PARP were up-regualted, while the expression level of Bcl-2 was reduced in SLPI-overexpressing SK-Hep-1 and HepG2 cells (Figure 5G, 5H). In SLPI-underexpressing BEL 7402 and HepG2 cells, the expression levels of Bax, PARP and cleaved PARP were down-regualted, while the expression level of Bcl-2 was up-regulated (Figure 5I, 5J).

### SLPI induced apoptosis of HCC cells via endoplasmic reticulum stress

**Figure 6:**
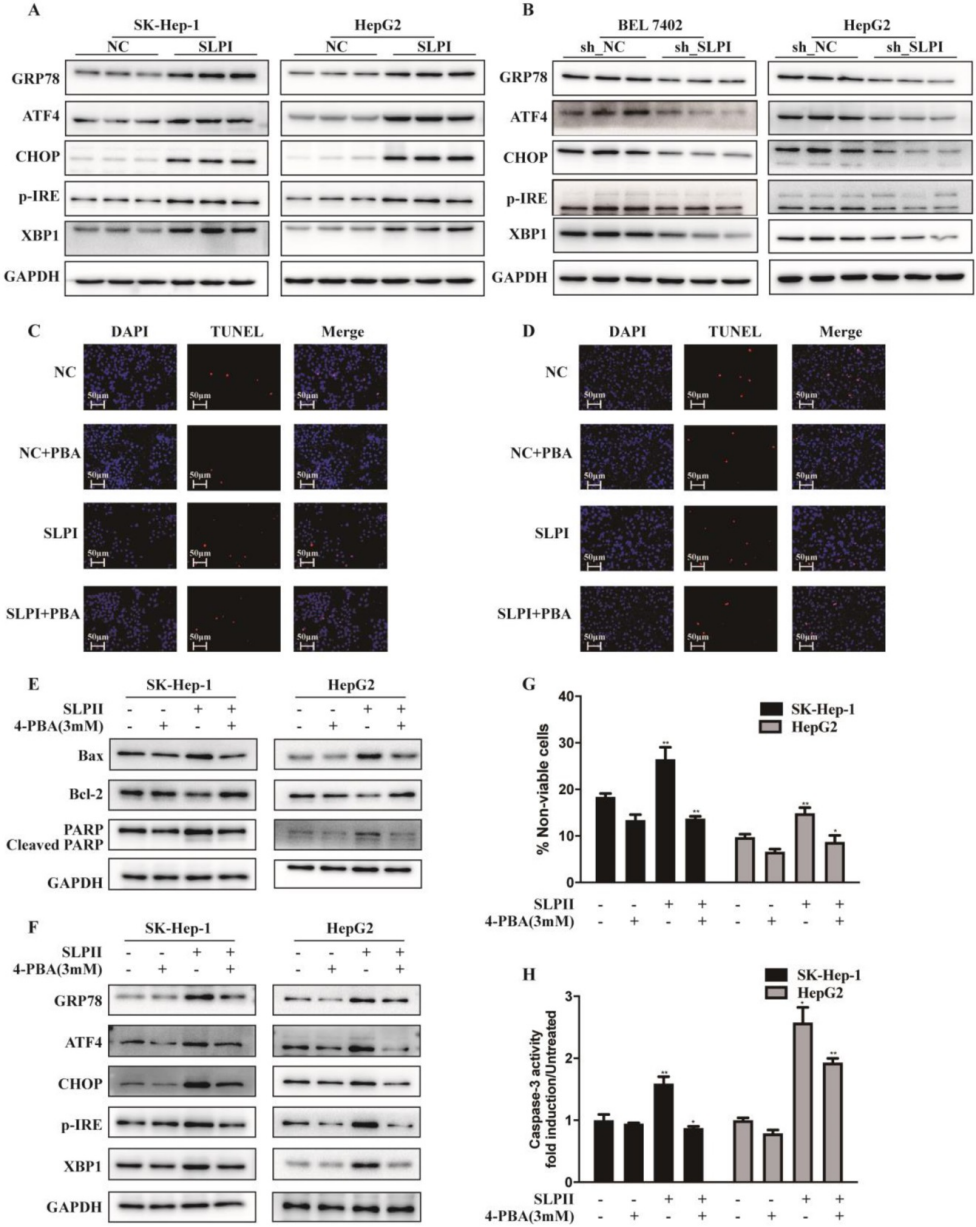
** SLPI induced HCC apoptosis via endoplasmic reticulum stress (ERS). A.**WB analysis of GRP78, ATF4, CHOP, p-IRE and XBP1 in SK-Hep-1 and HepG2 cells with SLPI overexpression. **B.** WB analysis of GRP78, ATF4, CHOP, p-IRE and XBP1 in BEL 7402 and HepG2 cells with SLPI knockdown. **C. D.** TUNEL assays were performed to determine the apoptosis of SK-Hep-1 and HepG2 cells with SLPI overexpression that treated by 4-Phenylbutyric acid (4-PBA). **E.** WB analysis of Bax, Bcl2, cleaved PARP and PARP in SK-Hep-1 and HepG2 cells with SLPI overexpression and 4-PBA treatment. **F.** WB analysis of GRP78, ATF4, CHOP, p-IRE and XBP1 in SK-Hep-1 and HepG2 cells with SLPI overexpression and 4-PBA treatment. **G.** Trypan blue staining analysis were performed to examine the apoptosis of SK-Hep-1 and HepG2 cells with SLPI overexpression and 4-PBA treatment. **H.** Caspase-3 activity analysis were performed to determine the apoptosis of SK-Hep-1 and HepG2 cells with SLPI up-regulation and 4-PBA treatment.

Considering the close connection between endoplasmic reticulum stress (ERS) and cell apoptosis, we further evaluated the effect of SLPI on several key molecules that are involved in the unfolded protein response including p-IRE-1, ATF-4, CHOP, XBP-1, GRP 78. These results indicated that the expression levels of GRP78, ATF4, CHOP, p-IRE and XBP1 were elevated in SLPI-overexpressing SK-Hep-1 and HepG2 cells (Figure 6A). While the expression levels of GRP78, ATF4, CHOP, p-IRE and XBP1 were decreased in SLPI-underexpressing BEL 7402 and HepG2 cells (Figure 6B).

Then we inhibited the activity of ERS signaling by 4-Phenylbutyric acid (4-PBA) treatment. In SLPI-overexpressing SK-Hep-1 and HepG2 cells, the results from TUNEL assay revealed that SLPI overexpression induced-apoptosis could be restored by 4-PBA treatment (Figure 6C, 6D). Consistently, up-regulation of Bax, PARP and cleaved PARP and down-regulation of Bcl2 could be rescued by 4-PBA treatment in SLPI-overexpressing SK-Hep-1 and HepG2 cells (Figure 6E). As for ERS associated proteins, up-regulation of GRP78, ATF4, CHOP, p-IRE and XBP1 could be restored by 4-PBA treatment in SLPI-overexpressing SK-Hep-1 and HepG2 cells (Figure 6F). In addition, trypan blue staining and caspase-3 activity determination further confirmed the inhibitory effect of 4-PBA on apoptosis induced by SLPI overexpression (Figure 6G, 6H). Together these results indicated that SLPI induced apoptosis of HCC cells through ERS.

### SLPI suppressed tumor growth and tumorigenesis *in vivo*

**Figure 7:**
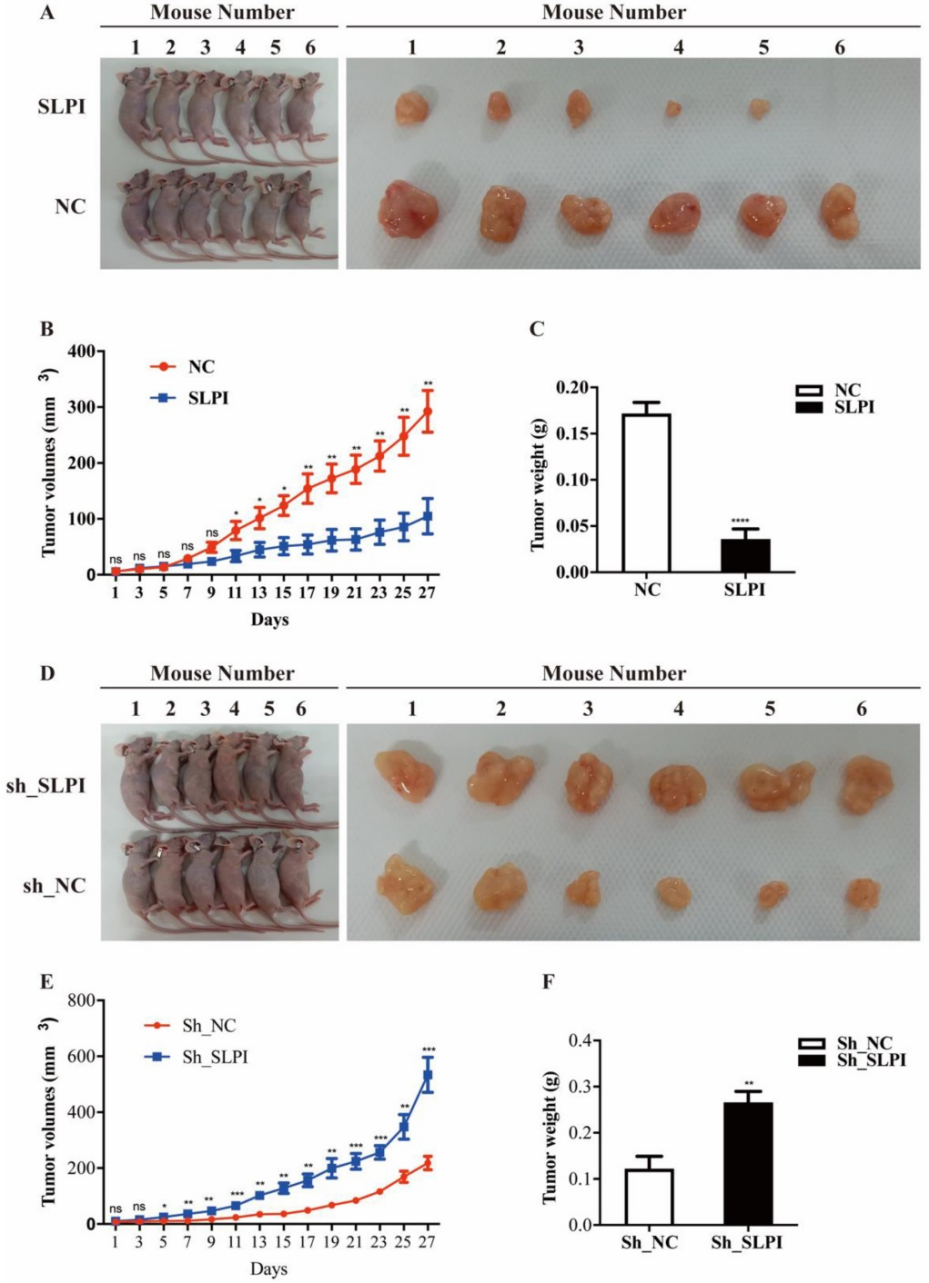
** SLPI suppressed tumor growth and tumorigenesis *in vivo*. A.** SLPI up-regulation inhibited growth of SK-Hep-1 xenograft tumors (n = 6 in each group). These tumors were formed by injection of SK-Hep-1 cells overexpressing SLPI or carrying a negative control. **B.** Tumor volumes were measured every 3 days. **C.** Average tumor weights of the xenografts were recorded. **D.** SLPI down-regulation promoted growth of BEL 7402 xenograft tumors (n = 6 in each group). These tumors were generated by injection of BEL 7402 cells with SLPI knockdown (sh_SLPI) or carrying a negative control (sh_NC).** E.** Tumor volumes were measured every 3 days. **F.** Average tumor weights of the xenografts were recorded.*P < 0.05, **p < 0.01, ***p < 0.001, ****p < 0.0001.

To further evaluate the anti-tumor effect of SLPI *in vivo*, we performed a xenograft formation experiment in BALB/C nude mice by using both SLPI-overexpressed SK-Hep-1 and SLPI-underexpressing BEL 7402 cells. As shown in figure 7A to 7C, SLPI-overexpressing SK-Hep-1 group showed a slower growth rate, smaller tumor volumes and lighter tumor weight compared with control group. However, tumors derived from SLPI-underexpressing BEL 7402 group developed faster, and both tumor volumes and tumor weight were significantly smaller than control group (figure 7D-7F). Taken together, these results further suggested the anti-tumor activities of SLPI toward HCC.

### The ERK/JNK/p38 signaling pathway was regulated by SLPI

**Figure 8:**
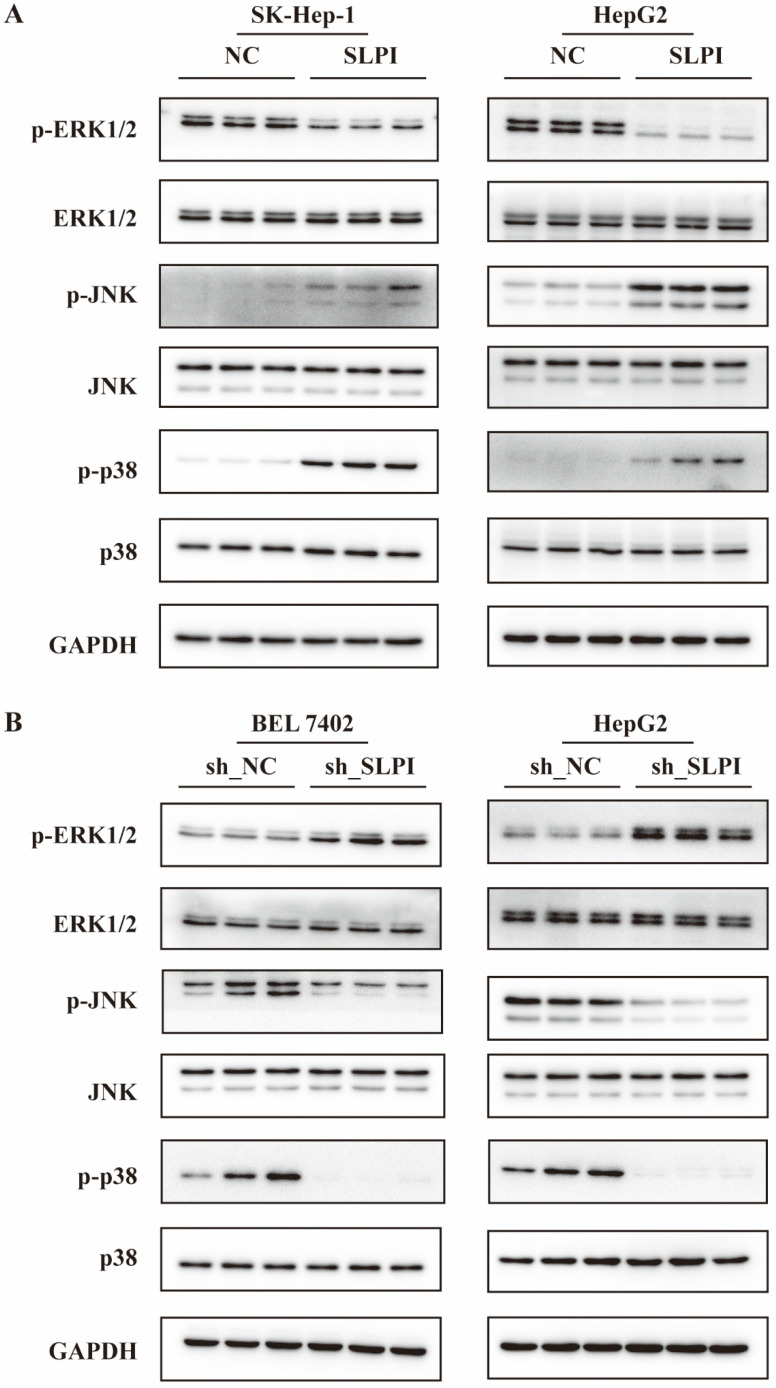
** The ERK/JNK/p38 signaling pathway was regulated by SLPI. A.** WB analysis of p- ERK1/2, total ERK1/2, p-JNK, total JNK, p-p38, total p-p38 in SK-Hep-1 and HepG2 cells with SLPI overexpression. Levels of p- ERK1/2, total ERK1/2, p-JNK, total JNK, p-p38, total p-p38 quantified on the basis of optical density and normalized to GAPDH levels in the same samples are shown below the WB results relative to average levels in NC cells. **B.** WB analysis of p- ERK1/2, total ERK1/2, p-JNK, total JNK, p-p38, total p-p38 in BEL 7402 and HepG2 cells with SLPI knockdown. Levels of p- ERK1/2, total ERK1/2, p-JNK, total JNK, p-p38, total p-p38 quantified on the basis of optical density and normalized to GAPDH levels in the same samples are shown below the WB results relative to average levels in NC cells.

From the data in figure 8A, overexpression of SLPI resulted in down-regulation of p- ERK1/2, and up-regulation of p-JNK, p-p38 in both SK-Hep-1 and HepG2 cells (figure 8A). Consistently, knockdown of SLPI gave rise to down-regualtion of p-JNK and p-p38, and up-regulation of p-ERK1/2. The results in this chapter indicated that SLPI appeared to modulate malignant biological behaviors of hepatoma cells and HCC progression via ERK/JNK/p38 signaling pathway.

## Discussion

Since the uncontrolled growth and metastasis of HCC cells, it becomes one of the most lethal malignancies at present time with high mortality and recurrence 17. For a long time, hepatitis B or hepatitis C virus is the main cause of liver cancer, but with the development of society and the improvement of life quality, obesity has become a global epidemic in the 21st century. Today, non-alcoholic steatohepatitis is recognized as a major driver of HCC 18. Multiple studies have shown that ER stress is involved in regulating non-alcoholic hepatitis-derived HCC. In addition to promoting the occurrence of the disease, ER stress is also involved in regulating the progression of HCC 6-8.

Previous studies have reported that SLPI as a inhibitor of serine protease is involved in many biological processes, such as antimicrobial action, antiviral activity, allergy reponses, autoimmunity, wound healing, cell proliferation, differentiation and apoptosis 10. A number of studies have reported that SLPI plays a signifcant role in the regulation of tumor progression and metastasis 15. The expression levels of SLPI vary according to different types of cancer. It has been reported that the expression of SLPI is up-regulated in some types of tumors such as non-small cell lung cancer, prostate cancer, inflammatory breast cancer and ovarian cancer, which could play a promoting role in tumor progression 19-21. Interestingly, for some other cancers, SLPI expression level is inversely correlated with tumor progression 20. For example, the expression of SLPI in advanced invasive lymphoma cells is significantly down-regulated compared with that in early non-invasive lymphoma cells 22, and low expression of SLPI has also been found in head and neck cancer 23, oral squamous cell carcinoma 24. These phenomena suggest that SLPI plays a protective effect in some type of tumors. In addition, Hu et al. found that SLPI is down-regulated in mouse breast cancer model with p53 deletion mutation 25. Researchers speculated that when SLPI is deficient in anti-protease function, the release of protease secreted by the tumor areas can't be suppressed to degrade the peri-cancerous tissues, thus promoting the spread of tumor cells. This process may be achieved by inhibiting the NF-kB pathway 25, 26. In our study, we first detected that SLPI was significantly down-regulated in HCC and its expression level was correlated with overall survival of HCC patients. Functional experiments showed that SLPI has the function of regulating the proliferation, migration and invasion capabilities of HCC cells through apoptosis pathway.

According to previous researches, SLPI, as a pleiotropic molecule, plays a crucial role in regulating a variety of biological processes. However, there have been no studies of the effects of SLPI on ER stress. A small number of studies have found that SLPI can be involved in the progression of ovarian cancer by regulating ERK MAPK signaling pathway 27, and it can also be involved in myocardial ischemia/reperfusion injury by regulating the p38 MAPK signaling pathway 28. ERK and p38 are both members of the MAPK signaling pathway family which plays an important role in regulating cellular stress response 29, 30. These findings suggest that SLPI may affect the progression of HCC by regulating ER stress. In our trials, we demonstrated that SLPI could enhance ER stress. Moreover, we need to further clarify whether MAPK signaling pathway is a necessary condition for SLPI-induced ER stress and apoptosis of liver cancer cells.

In conclusion, our study demonstrated that SLPI has anti-tumor effects both *in vivo* and *in vitro*. This study demonstrated for the first time that SLPI initiates the apoptotic response of HCC cells by activating ER stress and thereby regulating the proliferation, migration and invasion abilities of HCC cells, and ultimately affecting the progression of HCC. In addition, we found that the effect of SLPI on the progression of HCC is mediated by the MAPK signaling pathway. Hence, our data indicated that SLPI was a tumor suppressor and a pivotal biomarker for HCC therapy.

These conclusions are based on the responses of 135 patients who met operation criteria, and our results thus might not reflect the patients without indications for operation. Because of the incorrect contact information or the changes in contact information. We are now confirming our results in more patients and mice. Moreover, SLP1 is not a kinase and thus cannot activate MAPK signaling pathway directly. We are now exerting a tremendous fascination on its direct mechanism.

In summary, our data revealed the down-regulation of SLPI in HCC, and further indicated that SLPI played a key role in the ERS induced apoptosis of HCC by regulating ERK/JNK/p38 signaling pathway. Moreover, SLPI expression was associated with survival of HCC patients. Thus, SLPI may be a promising biomarker for HCC prognosis and a potential therapeutic target for HCC. Further studies of SLPI function are warranted in the future.

## Materials and methods

### Cell lines and cell culture

The human liver cancer cell lines PLC/PRF/5, SK-Hep-1, Huh7, BEL 7402, Hep3B, SMMC 7721, and HepG2 and normal human liver cells L02 were used. PLC/PRF/5, SK-Hep-1, Huh7, HepG2, Hep3B were purchased from the Library of Typical Culture of Chinese Academy of Sciences (Shanghai, China). L02 and BEL 7402 were purchased from Modern Analysis and Testing Center of Central South University (Changsha, China). SMMC 7721 cells were purchased from GeneChem Corporation (Shanghai, China). HepG2 and Hep3B were maintained in DMEM supplemented with 10% fetal bovine serum (FBS) and 1% penicillin/streptomycin. L02, SMMC-7721 and BEL 7402 cells were cultured in RPMI medium supplemented with 10% FBS and 1% penicillin/streptomycin. SK-Hep-1 and PLC/PRF/5 were cultured in MEM supplemented with 10% FBS and 1% penicillin/streptomycin. The above-mentioned cells were cultured in a humidified atmosphere containing 5% CO_2_ at 37°C.

### Packaging of SLPI lentivirus

Two lentivector-mediated SLPI short-hairpin RNA (shRNA1#: 5′-GCGTGACTTGAAGTGTTGCAT-3′, shRNA2#: 5′-CCTGACACTTGTGGCATCAAA-3′, and shRNA3#: 5′-GAGTCTGTCCTCCTAAGAAAT-3′) and negative control (NC) shRNA (TTCTCCGAACGTGTCACGT) were designed by GeneChem Corporation (Shanghai, China). Full-length sequences of SLPI cDNA were PCR amplified using the PrimeSTAR HS DNA polymerase (Takara, Tokyo, Japan) and subcloned into the GV341 plasmid (GeneChem, Shanghai, China). Lipofectamine TM 2000 (Invitrogen, Carlsbad, USA) was used to perform cell transfection.

### RNA extraction and real-time PCR

Total RNA from above-mentioned cells and human tissues were extracted by using Trizol reagent (Invitrogen, Carlsbad, CA, USA) according to the manufacturer's instructions. Then the 1000 ng of extracted RNA was used for cDNA synthesis (Takara, Tokyo, Japan). Next, RT-PCR was performed (Takara, Tokyo, Japan; Roche, Basel, Switzerland). Expression data were normalized to the geometric mean of the housekeeping gene GAPDH and calculated as 2-[(CT of indicated genes) - (CT of GAPDH)], where CT represents the threshold cycle for each transcript. The primers used were SLPI, forward: ′-GAGATGTTGTCCTGACACTTGTG-3′; SLPI reverse: 5′- AGGCTTCCTCCTTGTTGGGT-3′; GAPDH forward: 5′- GGAGCGAGATCCCTCCAAAAT-3′; and GAPDH reverse: 5′- GGCTGTTGTCATACTTCTCATGG-3′.

### Western blotting

Cells and tissues were harvested in RIPA lysis buffer (Beyotime, Shanghai, China) and heated for 10 min at 100°C. Protein concentrations were detected with a BCA protein assay kit (Beyotime, Shanghai, China). Equal quantities of protein were separated electrophoretically on 8%-10% SDS/polyacrylamide gels and transferred onto polyvinylidene difluoride membranes (Merck Millipore, Billerica, MA, USA). The membranes were probed with anti-SLPI rabbit antibody (Novus Biologicals, CO, USA), and antibodies to Bax, Bcl-2, PARP /Cleaved PARP, GRP78, ATF4, CHOP, phosphor-(p-)IRE, XBP1, phosphor-(p-)ERK1/2, ERK1/2, phosphor-(p-)JNK, JNK, phosphor-(p-)p38, and p38. The expression of each protein was determined with secondary antibodies (Zhongshan Golden Bridge, Beijing, China) and High-sig ECL Western Blotting Substrate (Tanon, Shanghai, China) according to the manufacturers' suggested protocols. The membranes were stripped and reprobed with anti-GAPDH antibody (Cell Signaling Technology, Beverly, MA USA) as a loading control.

### Immunohistochemistry

A total of 135 paraffin-embedded HCC samples were used. Clinicopathological classification and staging were determined according to the criteria of the American Joint Committee on Cancer 31. Patient consent and approval from the Institutional Research Ethics Committee were obtained for the use of these clinical materials for research purposes before the experiments were performed. The clinicopathological features of the patients are summarized in Table 1.

For the protein expression of SLPI in HCC tissues, we performed IHC. In brief, paraffin-embedded specimens were cut into 5 μm sections. The sections were deparaffinized and microwaved for antigenic retrieval. Then the sections were treated with an endogenous peroxidase blocker, then, incubated with goat serum to block nonspecific binding, and then with anti-SLPI overnight at 4°C.

After samples were washed in PBS three times, a secondary antibody was used for further incubation. Diaminobenzidene (Zhongshan Golden Bridge, Beijing, China) was used as a chromogen for color development. Then the slides were counterstained with hematoxylin, and dehydrated. Finally, the tissue sections were observed at 100×, 200×, and 400× magnification, and images were taken from six random fields of view. Quantitative analysis of IHC was performed with ImageJ and IHC Profiler software 32.

### Cell proliferation assays

The proliferation of HepG2, SK-Hep-1 and BEL 7402 cells were determined with CCK-8 assays (Biosharp, Hefei, China). These three cell lines were seeded in four 96-well plates. One of the plates was measured after growing for 4 hours, and the other three plates maintained for 24 hours, 48 hours, and 72 hours at 37°C, respectively. Then 100 μL fresh medium containing CCK-8 (10 μL) was put into each well and incubated at 37°C for 2 hours. The absorbance was measured at 450 nm.

### Colony formation assays

For colony formation assays, approximately 200 infected HepG2, SK-Hep-1 and BEL 7402 cells were seeded into six-well plates and cultured for 14 days at 37°C. After removal of the medium and washing with PBS, the cells were fixed in 4% paraformaldehyde for 20 minutes and then stained with Giemsa stain (Solarbio, Beijing, China). Only positive colonies (> 50 cells/colony) in the plates were counted and compared.

### Wound healing assays

First, “scratches” were created at the monolayer of infected HepG2, SK-Hep-1 and BEL 7402 cells. Second, capturing the images at the begaining and 72 hours. Third, the migration rates were quantified and compared according to the images.

### Transwell invasion assays

For transwell invasion assays, Transwell Biocoat Martrigel Invasion Chambers were purchased (Corning, NY, USA). Then about 200 μl infected HepG2, SK-Hep-1 and BEL 7402 cells were seeded into upper well of chambers without fetal bovine serum, and the lower chambers were filled with medium containing 15% serum. Next the chambers were incubated at 37℃ for 24 hours. Furthermore, the cells on the upper chamber membrane were wiped by soft cotton swabs. Finally, the cells underside the membrane was fixed in 4% paraformaldehyde for 20 minutes and then stained with crystal violet stain (Solarbio, Beijing, China). The number of invaded cells were counted and quantified under a microscope.

### Nude mouse tumor growth and tumorigenesis analysis

Female nude mice (BALB/c) were purchased from SLAC Laboratory Animal (Shanghai, China). The experiment was approved by the Institute Animal Ethical and Welfare Committee. The Mice were acclimated to 12 h light-dark cycle with 50% humidity and with free access to food and water for 2 weeks prior to experimentation. SLPI overexpressed SK-Hep-1 and SLPI down-regulated BEL 7402 cells were resuspended at 5 × 10^6^ cells/mL, and a 0.1 mL aliquot of cell suspension was injected subcutaneously into the right flank of the nude mice (n = 10/group). Tumor size was measured every 3 days. Tumor volumes were determined by external measurements and calculated according to the following equation: V= [L × W^2^] × π/6 (V = volume, L = length and W=width). 27 days later, the mice were sacrificed, and transplanted tumors were removed for assessment. All protocols in our animal experiments were in accordance with the Animal Ethical and Welfare Committee.

### Terminal deoxynucleotidyl transferase (TdT) dUTP Nick-End Labeling (TUNEL) assay

First, the infected HepG2, SK-Hep-1 and BEL 7402 cells were seeded on the climbing slices in the 24 well plates. Second, the aforementioned cells were fixed by paraformaldehyde for 30 minutes, and then washed by PBS. Third, the cell membranes were broken by 0.3% Triton X-100. Fourth, the cell climbing slices were incubated by TdT buffer for 60 minutes without light. Finally, these slices were stained by DAPI for 5 minutes, and then the images were taken by fluorescence microscope (550nm).

### Cell viable analysis

The cells were collected and resuspended with PBS. Then trypanblue dye solution was added into cell suspension for 5 minutes at room temperature. Next the 10µl of the final solution was added into the cell counting chamber. Trypanblue in blue representing the non-viable cells and the other marking the viable cells. (% non-viable cells = trypanblue staining cells/total counting cells ×100).

### Caspase3 activity assays

Caspase3 Activity Assay kits (Beyotime, Shanghai, China) were used to detect the activity of caspase 3. Briefly, the cells were collected and resuspended by prechilled lysis buffer, then centrifuged at 15000rpm, 4 ℃, for 5 15minutes. Second, the protein concentration of supernatant was dectected by using a Brandford Protein Assay kit (Beyotime, Shanghai, China). Third, the reaction buffer (0.2 mM Ac-DEVD-pNA) was added into lysates and a 2 hours' incubation at 37 ℃ was performed. Finally, the results were obtained by using microplate reader (Tecan, Zurich, Switzerland) at 405 nm.

### Statistical analysis

SPSS 23.0 (IBM, Armonk, NY, USA) and GraphPad Prism 7.0 software (La Jolla, CA, USA) were used for analysis. Chi-square tests were used to analyze the correlation between PES1 expression and the clinicopathological characteristics. Spearman's rank correlation coefficient was used to evaluate the bivariate correlations between the variables. Survival curves were plotted with the Kaplan-Meier method and compared through the log-rank test. The significance of various variables for survival was analyzed with the Cox proportional hazards model in univariate and multivariate analysis. Student's t tests or nonparametric tests were applied to analyze the differences between two groups. Differences among groups were calculated by one-way analysis of variance (ANOVA) with repeated measures. All data are shown as mean ± SEM from at least three independent experiments. p-values < 0.05 were considered significant.

## Figures and Tables

**Table 1:** Correlation between SLPI expression and clinicopathological characteristics of hepatocellular carcinoma patients.

	SLPI		
Characteristics	Low, no.cases(%)	High, no.cases(%)	*p* value
**Age**			
< 60	47 (34.8)	22 (16.3)	0.105
≥ 60	36 (26.7)	30 (22.2)	
**Gender**			
Male	70 (51.9)	42 (31.1)	0.592
Female	13 (9.6)	10 (7.4)	
**Etiology**			
HBV	75 (55.6)	45 (33.3)	0.592
HCV	2 (1.5)	1 (0.7)	
Alcohol	0 (0)	1 (0.7)	
Unknown	6 (4.4)	5 (3.7)	
**Imaging cirrhosis**			
Present	62 (45.9)	40 (29.6)	0.77
Absent	21 (15.6)	12 (8.9)	
**Distribution**			
Unilobar	78 (57.8)	52 (38.5)	0.071
Bilobar	5 (3.7)	0 (0)	
**AFP level,ng/mL**			
< 200	52 (38.5)	35 (25.9)	0.582
≥ 200	31 (23.0)	17 (12.6)	
**Pathological differentiation**			
Highly	9 (6.7)	5 (3.7)	0.15
Moderately	21 (15.6)	19 (14.0)	
Lowly	2 (1.5)	4 (3.0)	
Poorly	0 (0)	1 (0.7)	
Mixed differentiation	51 (37.8)	23 (17.0)	
**BCLC stage**			
0	8 (5.9)	4 (3.0)	0.687
A	61 (45.2)	41 (30.4)	
B	9 (6.7)	6 (4.4)	
C	5 (3.7)	1 (0.7)	
**Child-Pugh** (at randomization)			
A	75 (56.0)	50 (37.3)	0.509
B	6 (4.5)	2 (1.5)	
C	1 (0.7)	0 (0)	
**T classification**			
T1	58 (43.1)	42 (31.1)	0.492
T2	6 (4.4)	3 (2.2)	
T3	18 (13.3)	7 (5.2)	
T4	1 (0.7)	0 (0)	
**N classification**			
N0	79 (58.5)	51 (37.8)	0.386
N1	4 (3.0)	1 (0.7)	
**Metastasis**			
No	81 (60.0)	52 (38.5)	0.259
Yes	2 (1.5)	0 (0)	
**Method of diagnosis**			0.737
Imaging	82 (60.7)	51 (37.8)	
Biopsy	1 (0.7)	1 (0.7)	
**Vital states**(at flollow-up)			
Alive	33 (37.1)	32 (36.0)	0.016
Dead	14 (15.7)	10 (11.2)	

**Table 2:** Spearman analysis of correlation between SLPI and clinicopathological.

Variables	SLPI expression level	
	Spearman correlation	*p* value
Age	0.139	0.107
Gender	0.046	0.595
Etiology	0.047	0.604
Imaging cirrhosis	-0.025	0.772
Distribution	-0.155	0.072
Largest tumor size	-0.055	0.525
Method of diagnosis	0.029	0.739
AFP level, ng/mL	0.003	0.973
Pathological differentiation	-0.124	0.15
BCLC stage	-0.022	0.802
Child-Pugh(at randomization)	-0.092	0.289
T classification	-0.125	0.147
N classification	-0.075	0.39
Metastasis	-0.097	0.263
Vital states(at flollow-up)	0.218	0.024

**Table 3:** Univariate and multivariate analysis of various progonosis parameters in patients with hepatocellular carcinoma Cox-regression analysis.

	Univariate analysis			Multivariate analysis		
	p	Hazard ratio	95% Confidence interval	p	Hazard ratio	95% Confidence interval
**SLPI**	0.027	0.431	0.205-0.907	0.042	0.462	0.219-0.974
**T classification**	0.01	1.644	1.129-2.395	0.017	1.583	1.084-2.311
